# Supplementary material for: Phenotypic stasis with genetic divergence
Source: Peer Community J. Author manuscript; Available in PMC 2024 Sep 27. (PMC11434230; doi:10.24072/pcjournal.349)
Supplement: 1 [file NIHMS2024663-supplement-1.pdf]

Supplementary Information

0.1. Supplementary Figures

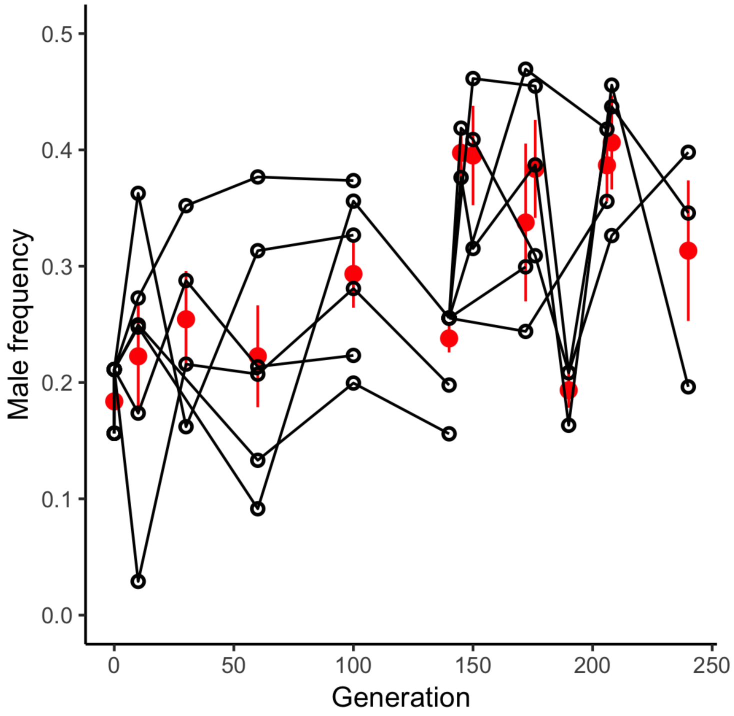

Figure S1 – Male frequencies during lab evolution. Males and hermaphrodite tracks were differentiated with a 30-trait classifier based on moments of size, shape and velocity-related traits derived from Multi-Worm Tracker metrics, and frequencies were estimated from 1s slices across movies. Empty circles indicate the estimates for each replicate population (between 1 and 6 at each time point), red circles the mean among replicate populations ($\pm$ standard error). During the first 100 generations of domestication, the estimates are similar to those obtained by directly counting the number of males (Teotónio et al., 2012).

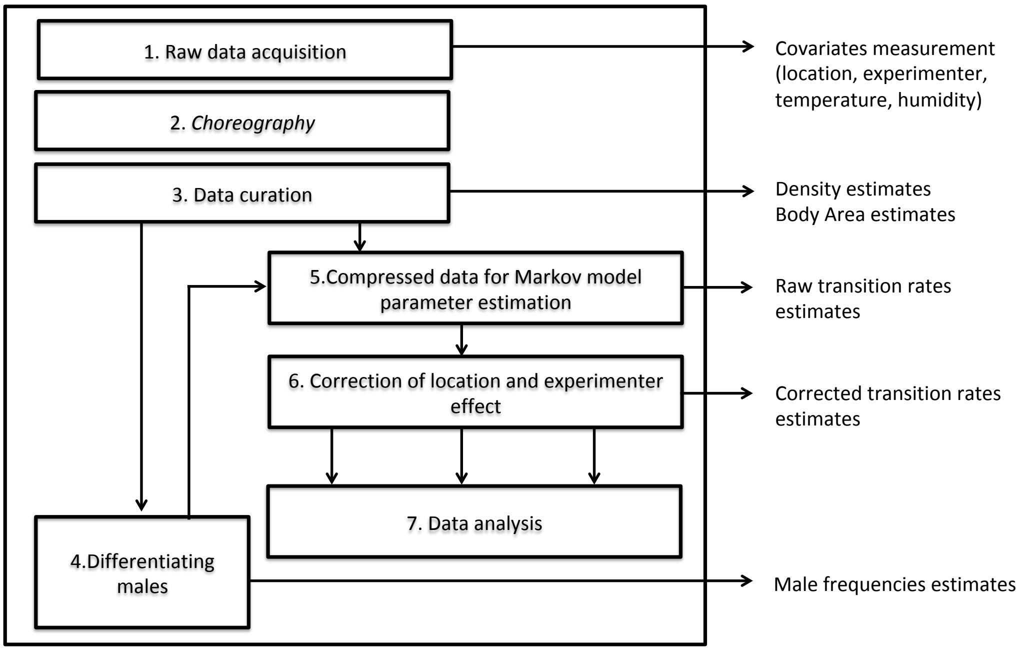

Figure S2 – Schematic of data acquisition and analysis pipeline.

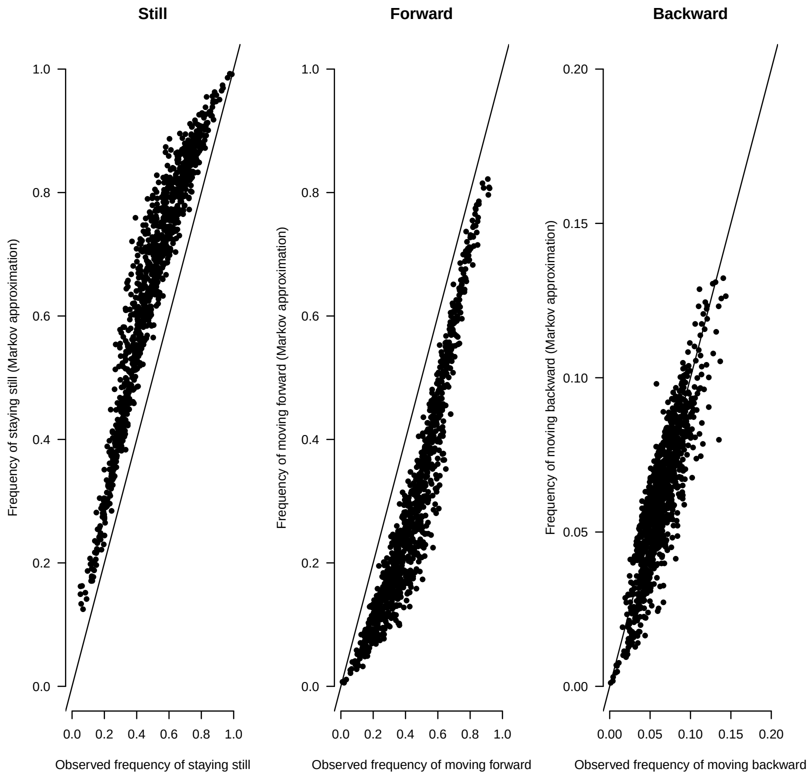

Figure S3 – Correlation between the observed frequencies of each of the three movement states and the predicted values from the Markov model. There is a consistent bias in the long term predictions due to violation of the memoryless assumption of the model. Some moving worms tend to remain in this state longer than expected on the long term, that is, they can be briefly interrupted but are more likely to resume movement than predicted.

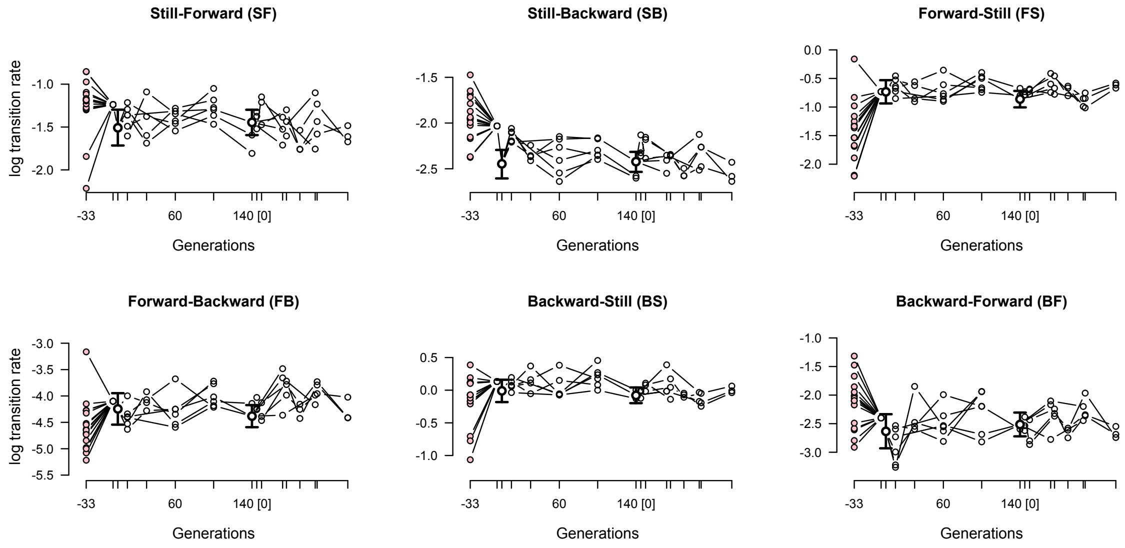

Figure S4 – Evolution of mean hermaphrodite transition rates. Each panel shows the evolution of a transition rate in the founders (pink dots) and during experimental evolution (white dots). At the beginning of the domestication and focal stages there was one ancestral population, shown by empty circles with 95% credible intervals, while 3-6 replicate populations were measured at each sampled time point indicated by tick marks.

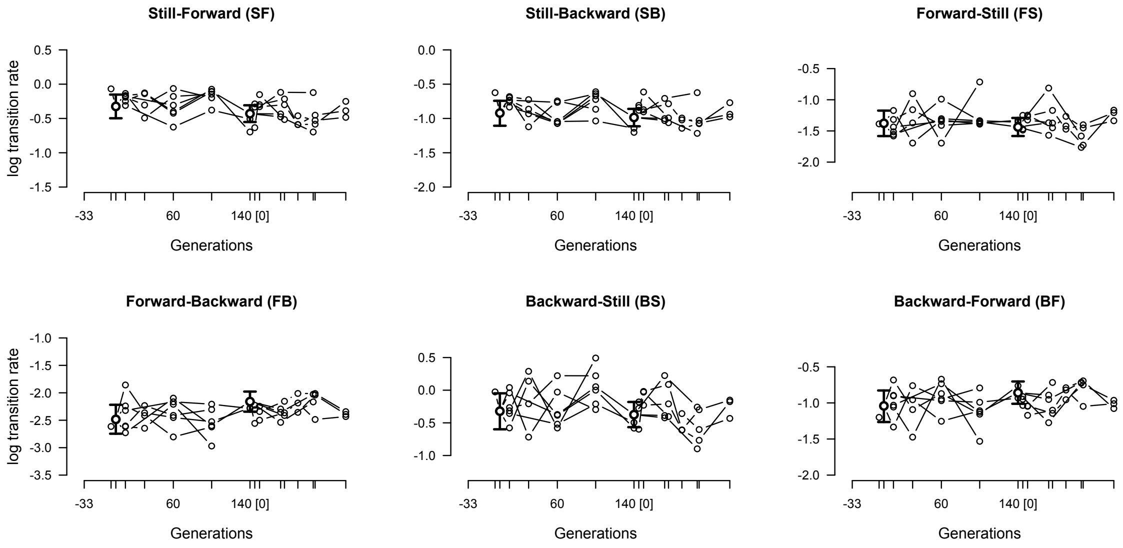

Figure S5 – Evolution of mean male transition rates, as in Figure S4. Note that the founder inbred lines do not have any males.

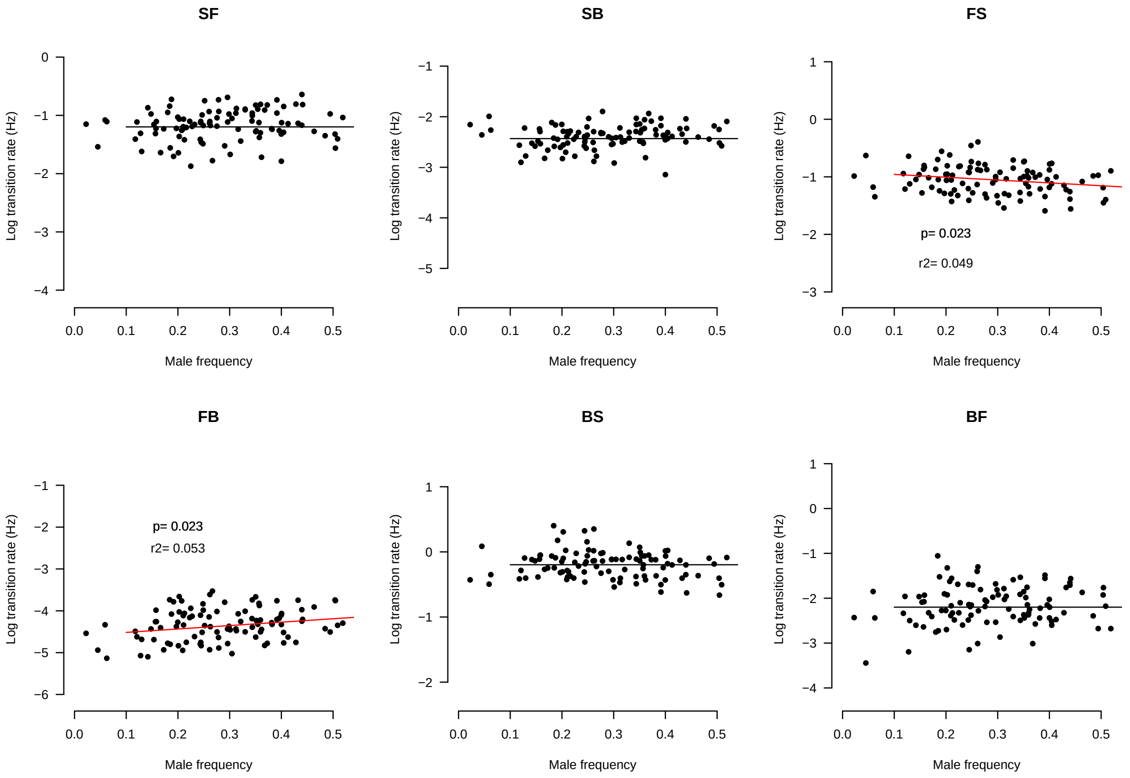

Figure S6 – The effects of males on hermaphrodite transition rates in the outbred populations during lab evolution. Each point shows the relation between transition rates and male frequency for each replicate population at a given time point during lab evolution. Red (black) lines show significant (non-significant) linear effects of male frequency on transition rates. For all regression models the coefficient of determination is extremely low (r^2).

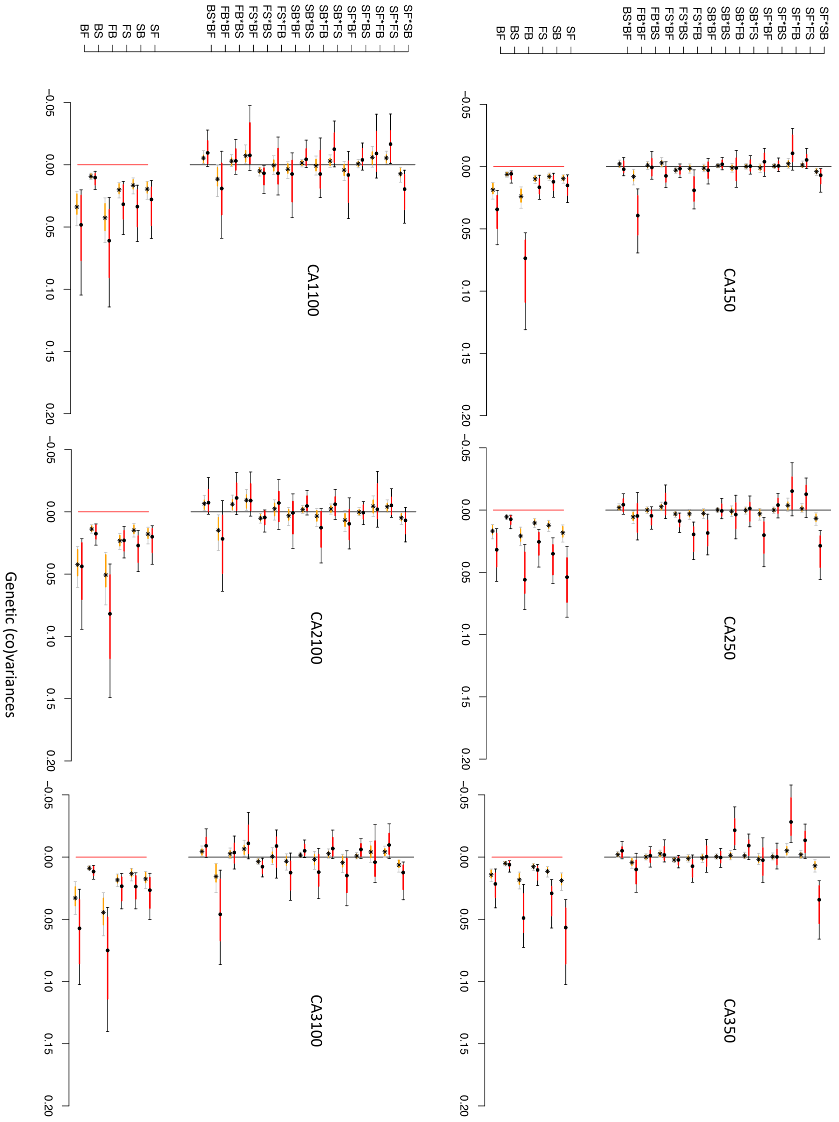

Figure S7 – Median genetic covariance and variance estimates of the CA[1-3] populations at generation 50 and generation 100 (dots), as well as random expectations (stars). Intervals are shown with the 95% and 83% credible intervals (black and red, grey and orange). Many of the covariance estimates do not differ from zero (95% interval) and covariance and variance median estimates do not generally differ from a null distribution obtained from random permutations of the phenotypic values (black dots are within grey bars), particularly for generation 100 populations. This is explained by a loss of genetic (co)variances with continued lab evolution after domestication, and not sampling a limited number of lines in each population, see Figure S7).

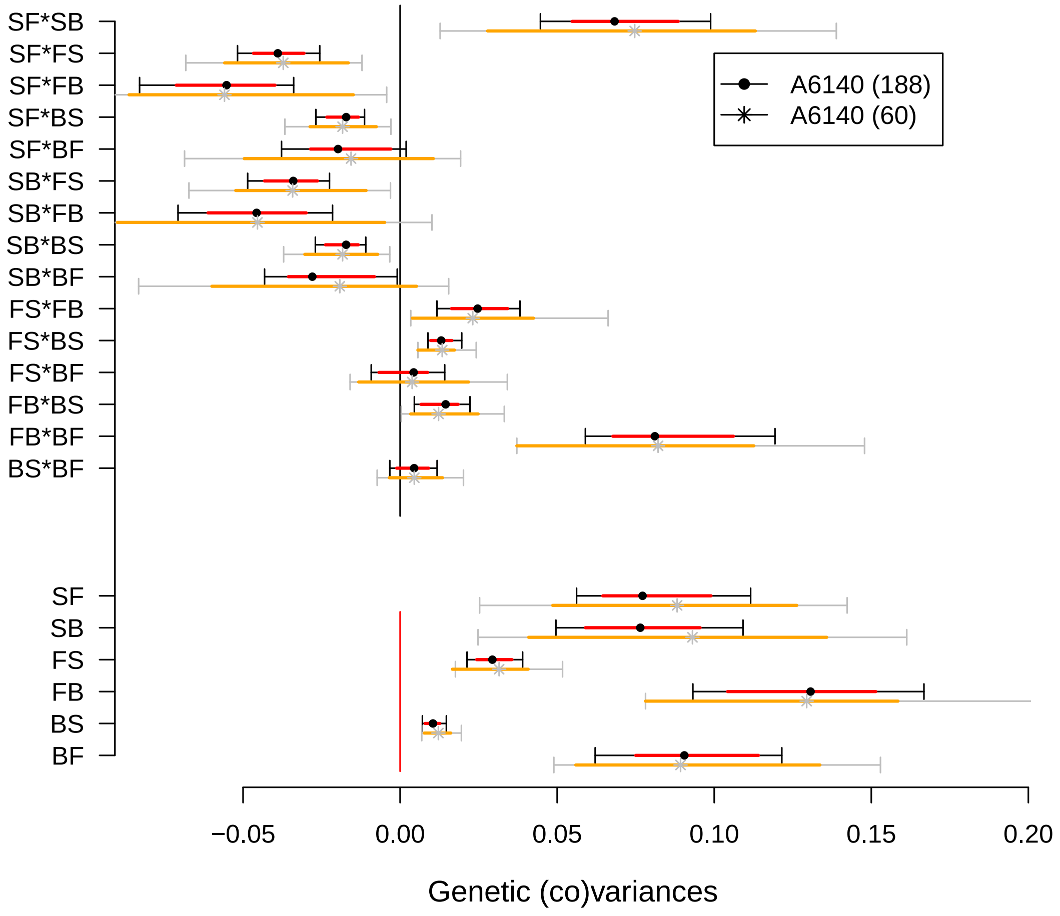

Figure S8 – **G**-matrix estimates of the 140-generation domesticated A6140 population. Black and red show the estimated genetic (co)variances using all inbred lines as in main Figure 3. Grey and orange show genetic (co)variances after downsampling to 60 inbred lines, approximately the minimum number of lines phenotyped in the CA[1-3] populations. Median estimates are similar between data sets, though with larger intervals in the subsampled estimates.

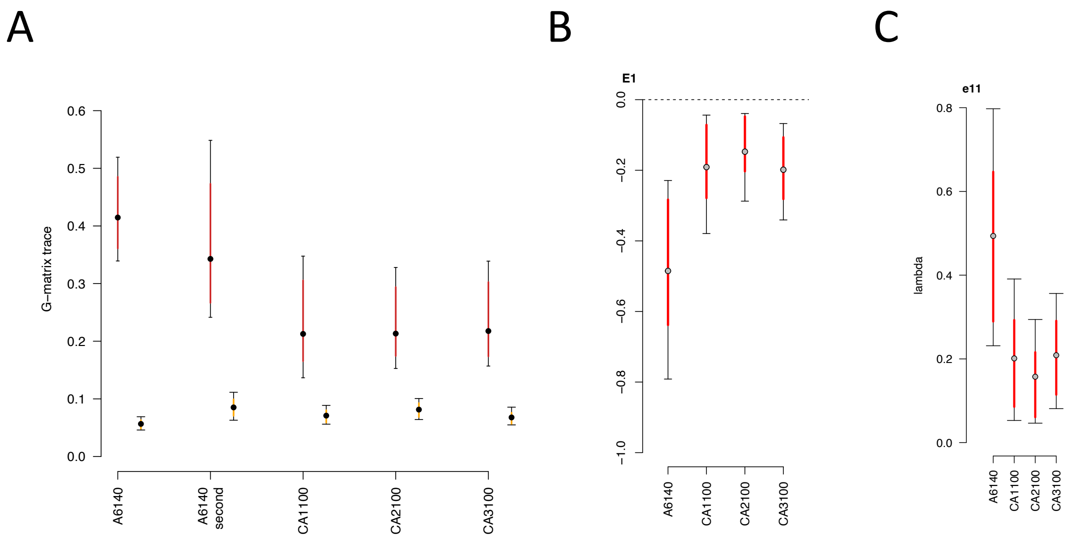

Figure S9 – Effect of the common garden assay on genetic (co)variances. **A.** Total amount of genetic variance computed as the sum of the **G**-matrix diagonal elements (trace). The amount of genetic variance in the two A6140 matrices from the two separate common gardens is similar. All observed posterior means differ from the null 95% posterior means (orange). **B.** The coordinates of the **G**-matrices in the space of the first eigentensor when comparing the A6140 and the CA[1-3]100 populations, all computed from the third common garden assay (see Methods). The absolute values of the **G**-matrix coordinates in each eigentensor represent its contribution to the difference between matrices. Coordinates with opposing signs indicate that the matrices contribute in opposing directions. **C.** Contribution of specific trait combinations to coordinated changes among **G**-matrices. Each panel shows the amount of genetic variance in the direction of the greatest variation among **G** (eigenvector of $\mathbf{E_1}$ only). Here, as in panels **A.** and **B.**, the results obtained from this second A6140 **G**-matrix are similar to when using the one from the first common garden assay.

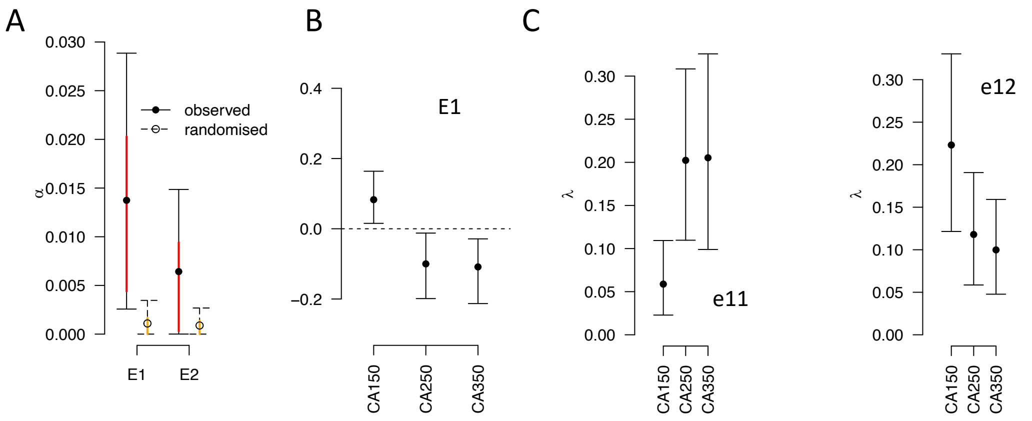

Figure S10 – Genetic differentiation. **A.** Eigentensor decomposition of variation among **G**-matrices of the CA[1-3]50 populations. The variance α_i associated with the ith eigentensor $\mathbf{E_i}$ is compared to a null permutation model where variation among matrices is due to sampling (see Methods). Here, only the first eigentensors is different from the null. **B.** The coordinates of the **G**-matrices in the space of the first eigentensor. The absolute values of the matrices coordinates in each eigentensor represent its contribution to the difference between matrices. Coordinates with opposing signs indicate that the matrices contribute in opposing directions. **C.** Contribution of specific trait combinations to coordinated changes among **G**-matrices. Each panel shows the amount of genetic variance in the direction of the greatest variation among **G** (eigenvector of $\mathbf{E_1}$ only).

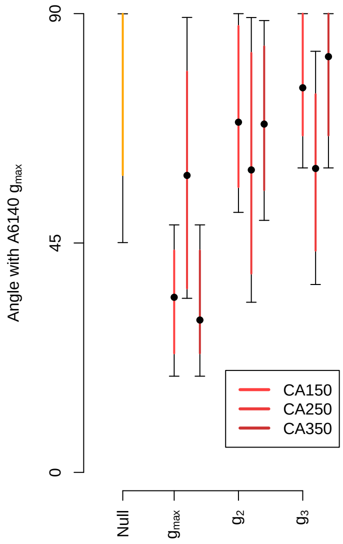

Figure S11 – The angle (Θ) between the first three eigenvectors of the evolved **G**-matrix (g_{max}, g_1 and g_3 of the CA[1-3]50 populations) with the A6140 g_{max}. Θ differs from the random expectations in CA150 and CA350 but not in CA250 showing genetic differentiation. Dots show the mean estimate with bars the 83% and 95% credible interval of the posterior **G**-matrix distribution. The null expectation was obtained by computing the angle between pairs of random vectors sampled from a uniform distribution.

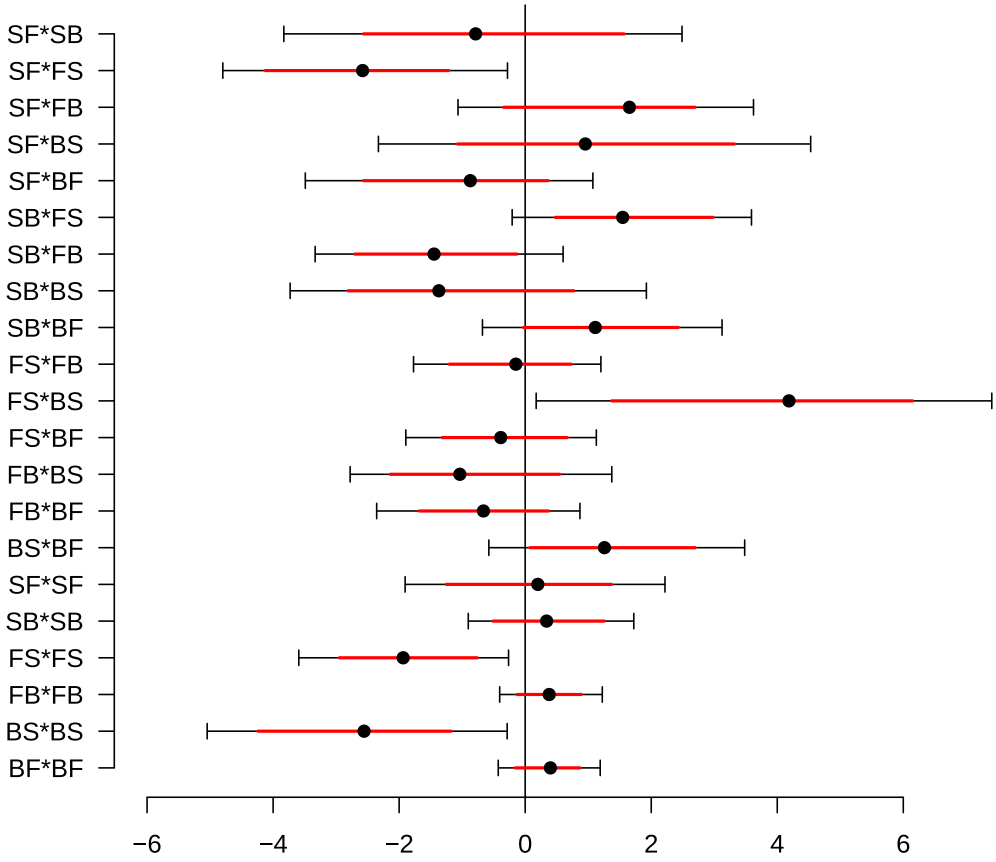

Figure S12 – Quadratic selection coefficients. The partial regression coefficients of fertility on trasition rates estimated by Bayesian inference. Each row shows the mode (dot), and 83% and 95% credible intervals (red bar and line bars, respectively) of the posterior distributions. The top 15 rows show coefficients of correlated selection between two transition rates, the bottom 6 rows show coefficients of stabilizing or disruptive selection on each transition rate.

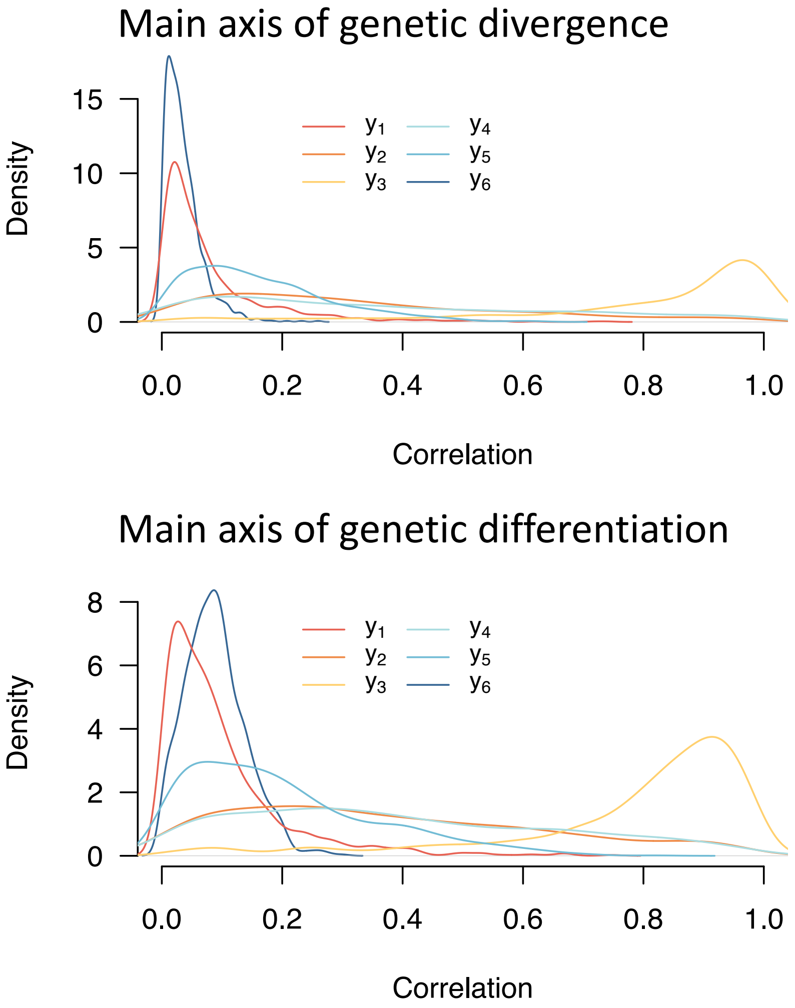

Figure S13 – Alignment of **G**-matrices divergence and differentiation with the quadratic selection surface. Shown is the density distributions of Pearson product moment correlations between the first eigenvector e_{11} of **E1** measured for divergence (between A6140 and CA[1-3]100, top panel) and for genetic differenciation (measured among CA[1-3]50, bottom panel). The density distributions are obtained from 1000 sampling in the posterior distribution of the γ matrix.

0.2. Supplementary Tables

Population name	Generations	Stage
OF5, A0	-5,0	Ancestral population
A1,A2,A3	10;30;60;100	Domestication
A4	10;30;40;60;100	
A5,A6	10;40;60;100	
CA1, CA2, CA3	5;10;36:50;68;100	Focal
CA4, CA5	32;66	
CA6	32	

Table S1 – Identity of the phenotyped populations shown in Figure 1C. The first column presents the population names, composed of a prefix followed by a replicate number. The prefixes "A" and "CA" stand for domestication and focal stages respectively. "CA" populations were all started from the A6 population at generation 140 (A6140) and the generation number restarted at 0. OF5 populations was frozen 5 generations before the A0 during hybridization.

Transition rate	Sex	Chisq	P value	P adjusted
SF	H	0.542	0.462	0.799
SB	H	0.715	0.398	0.799
FS	H	0.389	0.533	0.799
FB	H	3.077	0.079	0.799
BS	H	0.037	0.847	0.924
BF	H	1.810	0.179	0.799
SF	M	0.414	0.520	0.799
SB	M	0.647	0.421	0.799
FS	M	0.542	0.462	0.799
FB	M	0.053	0.817	0.924
BS	M	0.001	0.980	0.980
BF	M	0.082	0.774	0.924

Table S2 – Phenotypic stasis: Results of anova LRT χ_1^2 tests for directional changes in mean transition rates in hermaphrodites (H) and males (M), during the 240 generations of lab evolution. Corrected P values for multiple comparisons were obtained with the Benjamini-Hochberg method. Transition rates notation XY stands for transition from trait X to Y, S: Still, F: Forward and B: Backward.

Transition rates	Chisq	P values	P adjusted
SF	5.445	0.020	0.118
SB	1.319	0.251	0.419
FS	0.107	0.743	0.860
FB	1.443	0.230	0.419
BS	1.170	0.279	0.419
BF	0.031	0.860	0.860

Table S3 – Inbreeding effects: Results of anova LTR χ_1^2 testing for mean phenotypic differences between the mean of the inbred lines and the mean of the A6140 population from which they were derived. Corrected P values for multiple comparisons were obtained with the Benjamini-Hochberg method. Transition rates notation XY stands for transition from trait X to Y, S: Still, F: Forward and B: Backward.

	A6140 population					
	g_{max}	g_2	g_3	g_4	g_5	g_6
Eigenvalues	0.263	0.105	0.022	0.01	0.005	0.003
HPD lower	0.196	0.074	0.016	0.007	0.003	0.002
HPD upper	0.348	0.156	0.03	0.014	0.006	0.003
Proportion	0.645	0.257	0.054	0.025	0.012	0.007
Trait loadings:						
SF	-0.438	0.474	0.086	0.286	-0.539	-0.451
SB	-0.423	0.444	-0.488	0.198	0.501	0.31
FS	0.214	-0.315	-0.176	0.717	0.297	-0.472
FB	0.629	0.383	-0.596	-0.162	-0.236	-0.143
BS	0.112	-0.127	-0.074	0.533	-0.488	0.666
BF	0.419	0.563	0.602	0.234	0.276	0.119

Table S4 – Eigendecomposition of the A6140 G-matrix. Transition rates notation XY stands for transition from trait X to Y, S: Still, F: Forward and B: Backward.

	Gamma					
	γ_1	γ_2	γ_3	$\gamma 4$	γ_5	γ_6
Eigenvalues	4.904	0.256	0.02	-0.172	-1.489	-10.256
HPD lower	0.239	0.008	-0.14	-1.031	-3.456	-18.076
HPD upper	12.295	1.094	0.234	0.036	-0.46	-3.9
Proportion	0.287	0.015	0.001	0.01	0.087	0.6
Trait loadings:						
SF	0.527	-0.429	-0.212	0.378	0.544	-0.234
SB	-0.479	-0.167	-0.744	-0.251	0.3	0.192
FS	-0.296	0.282	0.274	-0.276	0.536	-0.628
FB	0.514	-0.132	0.026	-0.839	0.039	0.112
BS	-0.11	0.016	0.447	0.062	0.548	0.695
BF	-0.359	-0.831	0.356	-0.104	-0.154	-0.135

Table S5 – Eigendecomposition of the γ G-matrix. Transition rates notation XY stands for transition from trait X to Y, S: Still, F: Forward and B: Backward.
